# Serum norethisterone (NET) levels in NET-enanthate (NET-EN) injectable contraception users substantially interfere with testosterone immunoassay measurements and confound interpretation of biological outcomes

**DOI:** 10.1186/s40834-025-00388-x

**Published:** 2025-08-19

**Authors:** Chanel Avenant, Johnson Mosoko Moliki, Alexis J. Bick, Sigcinile Dlamini, Mandisa Singata-Madliki, G. Justus Hofmeyr, Pai-Lien Chen, Karl-Heinz Storbeck, Donita J. Africander, David W. Erikson, Janet P. Hapgood

**Affiliations:** 1 Department of Molecular and Cell Biology, University of Cape Town, Upper Campus, Private Bag, Rondebosch, Cape Town, Western Cape 7700 South Africa; 2 Effective Care Research Unit, Eastern Cape Department of Health/Universities of the Witwatersrand and Fort Hare, East London, South Africa; 3 Walter Sisulu University, East London, South Africa; 4 Department of Obstetrics and Gynecology, University of Botswana, Gaborone, Botswana; 5 Family Health International (FHI) 360, Durham, NC USA; 6 Department of Biochemistry, Stellenbosch University, Stellenbosch, South Africa; 7 Endocrine Technologies Core, Oregon National Primate Research Center, Oregon Health and Science University, Beaverton, OR USA; 8 Institute of Infectious Disease and Molecular Medicine, University of Cape Town, Cape Town, South Africa

**Keywords:** Norethisterone enanthate (NET-EN), Depo medroxyprogesterone acetate intramuscular (DMPA-IM), Testosterone, Testosterone quantification, Chemiluminescent microparticle immunoassay (CMIA), Ultra-high performance liquid chromatography tandem mass spectrometry (UHPLC-MS/MS), Injectable contraceptives

## Abstract

**Background:**

The progestin norethisterone (NET), which is structurally related to testosterone, and its enanthate form (NET-EN), are used in contraception in women. Oral NET has been shown to interfere with testosterone measurements by some chemiluminescence microparticle immunoassays (CMIA). However, whether serum NET in NET-EN users interferes with these assays is unknown.

**Methods:**

Serum samples were obtained from women randomized to the injectable contraceptives NET-EN or depo medroxyprogesterone acetate intramuscular (DMPA-IM) in a clinical trial conducted in South Africa. Testosterone concentrations were compared after measurement by Abbott Architect CMIA and ultra-high performance liquid chromatography tandem mass spectrometry (UHPLC-MS/MS), from matched samples collected at baseline (D0) and 25 weeks (25W) after initiation.

**Results:**

At 25W, testosterone concentrations in the NET-EN arm were significantly higher (271%) using the CMIA compared to the UHPLC-MS/MS method. Contrary to the UHPLC-MS/MS results showing a significant decrease in testosterone concentrations in the NET-EN arm from D0 to 25W, a significant increase was determined by CMIA. Conversely, in the DMPA-IM arm at 25W, no significant difference in testosterone concentrations between the two methods was detected, and both methods showed a significant decrease in testosterone from D0 to 25W.

**Conclusions:**

We show for the first time that physiological concentrations of NET in premenopausal NET-EN users interfere with testosterone quantification using a CMIA method. The degree of interference is much higher and occurs at lower concentrations of NET than has previously been reported for oral NET and confounds the biological outcome of NET-EN use on testosterone concentrations, individually and relative to DMPA-IM.

**Trial registration:**

The WHICH trial was retrospectively registered with the Pan African Clinical Trials Registry (PACTR 202009758229976).

**Supplementary Information:**

The online version contains supplementary material available at 10.1186/s40834-025-00388-x.

## Background

Testosterone is one of the major androgens in premenopausal women and plays an important role in female sexual function, cognition, metabolism, mood, and cardiovascular health [1]. In women, increased testosterone concentrations (hyperandrogenism) are routinely observed in clinical conditions such as polycystic ovary syndrome (PCOS), congenital adrenal hyperplasia, and in androgen‐secreting tumours of the ovary or adrenal gland [2]. Decreased testosterone concentrations (hypoandrogenism) are associated with reduced sexual desire and libido [3], which may impact women’s exposure to sexually transmitted infections, including HIV acquisition. Changes in testosterone levels in women on contraception have been proposed to play a role in changes in sexual behavior [4]. Accurate quantification of testosterone concentrations in women is therefore crucial for understanding the potential effects on health and behavior outcomes, which is of particular importance in sub-Saharan Africa, a region with high HIV/AIDS incidence and prevalence among young women and girls [5].

Injectable contraceptives, predominantly intramuscular (IM) depo medroxyprogesterone acetate (DMPA-IM), a three-monthly injectable contraceptive containing 150 mg MPA, are the most widely used contraceptive methods in sub-Saharan Africa [6]. A two-monthly injectable contraceptive, norethisterone enanthate (NET-EN), containing 200 mg NET-EN, is used in select sub-Saharan African countries, including widely in South Africa [7].

The Women’s Health, Injectable Contraceptive, and HIV (WHICH) clinical trial conducted at clinical sites in Durban and East London in South Africa, randomized HIV-negative women aged 18 to 40 years to DMPA-IM (n = 262) or NET-EN (n = 259) [8]. Serum samples were collected from 435 women (215 DMPA-IM; 220 NET-EN) at baseline (before receiving injectable contraception) (Day 0, D0) and one week after the 24-week injection, i.e. at 25 weeks (25W), the time most likely to reflect peak serum progestin concentrations [9–11]. In a secondary study, the effects of these contraceptives on endogenous serum testosterone concentrations were determined using ultra-high performance liquid chromatography tandem mass spectrometry (UHPLC-MS/MS) [12]. While both contraceptives substantially reduced testosterone concentrations, NET-EN did so significantly more than DMPA-IM [12]. This decrease in testosterone with DMPA-IM use is consistent with other reports for DMPA-IM or subcutaneous DMPA (DMPA-SC) use, both showing a decrease in testosterone measured using immunoassays [13, 14]. However, the decrease in testosterone measured by UHPLC-MS/MS with NET-EN use [12] appears inconsistent with a study by Lawrie et al*.,* where no significant difference was detected in testosterone quantified by a chemiluminescent microparticle immunoassay (CMIA) in women 6 weeks after a single NET-EN injection, compared to women from the placebo arm [15]. To our knowledge, no other studies have used CMIA to measure testosterone in NET-EN users. Furthermore, no other study has used liquid chromatography-tandem mass spectrometry (LC–MS/MS) to measure testosterone concentrations in women on DMPA-IM or NET-EN, making it difficult to directly compare our testosterone data or to determine whether the different results obtained with NET-EN in our study and that of Lawrie et al*.* are due to the different methods used to measure testosterone (UHPLC-MS/MS vs CMIA).

It is well-documented that LC–MS/MS and gas chromatography–mass spectrometry methods are more sensitive and accurate in determining testosterone concentrations, specifically at low concentrations, as compared to immunoassay methods [16–19]. Testosterone concentrations in healthy women are substantially lower than those of men, typically ranging from 0.06–1.68 nmol/L when determined by LC–MS/MS methods [20–23]. Besides having a reduced sensitivity at low concentrations, immunoassays for testosterone are also prone to interference by multiple structurally related steroids, due to the limited specificity of antibodies (Reviewed in [24]). This is particularly so for 19-nortestosterone and several other endogenous and synthetic steroids, including the progestins norgestrel (ethyl-17α-ethynyl-19-nortestosterone) and norethisterone/norethindrone (19-nor-17α-ethynyltestosterone, NET), but not for MPA (6α-methyl-17α-hydroxyprogesterone acetate) [25]. NET and MPA are the active contraceptive progestins detected in the serum of NET-EN and DMPA-IM users, respectively. Apart from the potentially interfering compounds mentioned in the CMIA package insert, independent laboratories have also reported interference by dehydroepiandrosterone sulphate (DHEA-S) [26–29]. Jeffery et al. used several different commercially available immunoassays to measure testosterone concentrations in 3 premenopausal women using oral NET daily and further investigated possible interference with relatively high concentrations of exogenously added NET (50.3 or 100 nmol/L) [28]. At testosterone concentrations of ~ 1.33 nmol/L, as determined by LC–MS/MS, they reported interference by exogenously added NET for two immunoassays, with little to no detectable interference with the Abbott Architect CMIA [28]. In another study, Rowe and Rabet used the Abbott Architect 2nd generation CMIA to measure testosterone and found that the concentrations increased by ~ 1.5-, 3.2-, or 5.0-fold with the exogenous addition of 10, 50, or 100 nmol/L NET, respectively [29], although they did not verify their results by LC–MS/MS. The Abbott Architect 2nd generation testosterone CMIA package insert states that 10 nmol/L (2.98 ng/mL) NET has 0.7% cross-reactivity at testosterone concentrations of 2.4 nmol/L, resulting in a testosterone concentration difference of 0.07 nmol/L [25] or about 3%, suggesting minimal interference at this concentration. However, information on the degree of interference by NET at testosterone concentrations lower than 2.4 nmol/L is not provided [25].

The two studies mentioned above demonstrated that relatively high concentrations of exogenously added NET can interfere with testosterone measurements by immunoassays [28, 29]. Similarly, in a small study of only three women, Jeffery et al*.* showed that the use of oral NET can also interfere with testosterone measurements by immunoassays [28]. However, to date, there is no information available on whether physiologically relevant concentrations of NET in the serum of NET-EN users interfere with endogenous testosterone quantification by immunoassays in premenopausal women. In addition, while it has been shown that chromatography-mass spectrometry methods are more sensitive and accurate in determining testosterone concentrations, as compared to immunoassay methods, immunoassays are still widely used by clinicians worldwide [13, 30–34]. A recent survey on testosterone measurements in laboratories across Europe indicated that about 74% of all laboratories used immunoassays to measure testosterone [30]. Furthermore, a recent systematic review and diagnostic meta-analysis on evaluating androgen measurement in diagnosing PCOS in women found that about 60% of the studies used immunoassays to determine testosterone concentrations [31]. While no survey information about the percentage use of immunoassays to determine testosterone concentrations in sub-Saharan African countries is available, determining whether physiologically relevant concentrations of NET in the serum of NET-EN users interfere with endogenous testosterone quantification by immunoassays is particularly important given the wide use of NET-EN in select sub-Saharan African countries [7]. We report here the quantification of testosterone concentrations at D0 and 25 W, by immunoassay using the Abbott Architect 2nd generation testosterone assay compared to data obtained by UHPLC-MS/MS [12] in donor-matched serum samples.

## Materials and methods

### Primary study, ethics, and biosafety

Evaluating testosterone concentrations was a secondary study of the open-label randomized WHICH clinical trial, which aimed to evaluate estradiol concentrations and menstrual, psychological, and behavioral measures relevant to HIV risk. The WHICH study protocol and primary paper have been reported elsewhere [8]. The study was registered with the Pan African Clinical Trials Registry (PACTR 202009758229976). The application for additional tests on stored biological samples was approved by the University of Cape Town’s Faculty of Health Sciences Human Research Ethics Committee (HREC REF no. 664/2018). All women provided informed, written consent to authorize study participation and storage of samples. All researchers performing assays on archived samples or analyzing the data were blinded to the study arm and did not have access to any information that could identify participants. Archived samples were processed between 01 November 2021 and 17 August 2022.

### Study design and sample collection

Briefly, HIV-negative women (aged 18–40), seeking contraception at the East London and Mdantsane public health clinics and hospitals (Frere and Cecilia Makiwane Hospitals) (ECRU), South Africa, and the research site of the University of the Witwatersrand MatCH Research Unit (MRU), based in Durban, KwaZulu-Natal, South Africa, were randomized to 150 mg DMPA-IM 12-weekly or 200 mg NET-EN IM eight-weekly. Exclusion criteria were participants who reported receiving DMPA-IM in the previous six months or NET-EN in the previous four months via self-report, were living with HIV, or were using or intending to use medication that might have interfered with biological measurements, such as steroids or drugs affecting renal function, such as pre-exposure prophylactic drugs. Blood samples were collected at D0 and 25 W, i.e., about seven days after the 24-week progestin injection, and serum was separated and stored at −80 °C.

### Testosterone measurements by immunoassay

Testosterone concentrations in 435 women (220 NET-EN; 215 DMPA-IM), D0 and 25 W, were measured at Neuberg Global Laboratories (Durban, KwaZulu Natal, South Africa) by CMIA (Abbott Architect 2nd Generation Testosterone: sensitivity or limit of quantification (LOQ) ≤ 0.15 nmol/L; Precision ≤ 10% coefficient of variation (CV) at testosterone concentrations ≥ 0.5 nmol/L).

### UHPLC-MS/MS quantification of testosterone, NET, and MPA

Testosterone, NET, and MPA were quantified in the serum samples from 435 women at D0 and 25 W by UHPLC-MS/MS at the Central Analytical Facility at Stellenbosch University in Stellenbosch, South Africa [10, 12]. Based on sample availability and budget constraints, for a small subset of 58 women (27 NET-EN; 31 DMPA-IM) randomly selected, matching serum samples were sent to the Endocrine Technologies Core (ETC) facility at Oregon National Primate Research Center/Oregon Health & Science University (OHSU) in the U.S.A. and testosterone was measured by UHPLC-MS/MS, as previously described [35].

Henceforth, the testosterone data measured at OHSU will be referred to as OHSU UHPLC-MS/MS, to distinguish it from UHPLC-MS/MS data determined at Stellenbosch University. The OSHU and Stellenbosch facilities have different UHPLC-MS/MS instruments (Stellenbosch: Xevo TQ-S triple quadrupole mass spectrometer; OHSU: Shimadzu Nexera-LCMS-8050 instrument), and each facility used its own previously established methodology to accurately determine low testosterone concentrations. These differences include the amount of serum used (Stellenbosch: 500 μL; OHSU: 200 µL), extraction solvent (Stellenbosch: methyl tert butyl ether; OHSU: dichloromethane), and chromatography columns (Stellenbosch: ACQUITY UPLC HSS T3 1.8 μm 2.1 mm × 50 mm; OHSU: Raptor 2.7 µm Biphenyl 50 mm × 2.1 mm).

### Data analysis

**Fig. 1:**
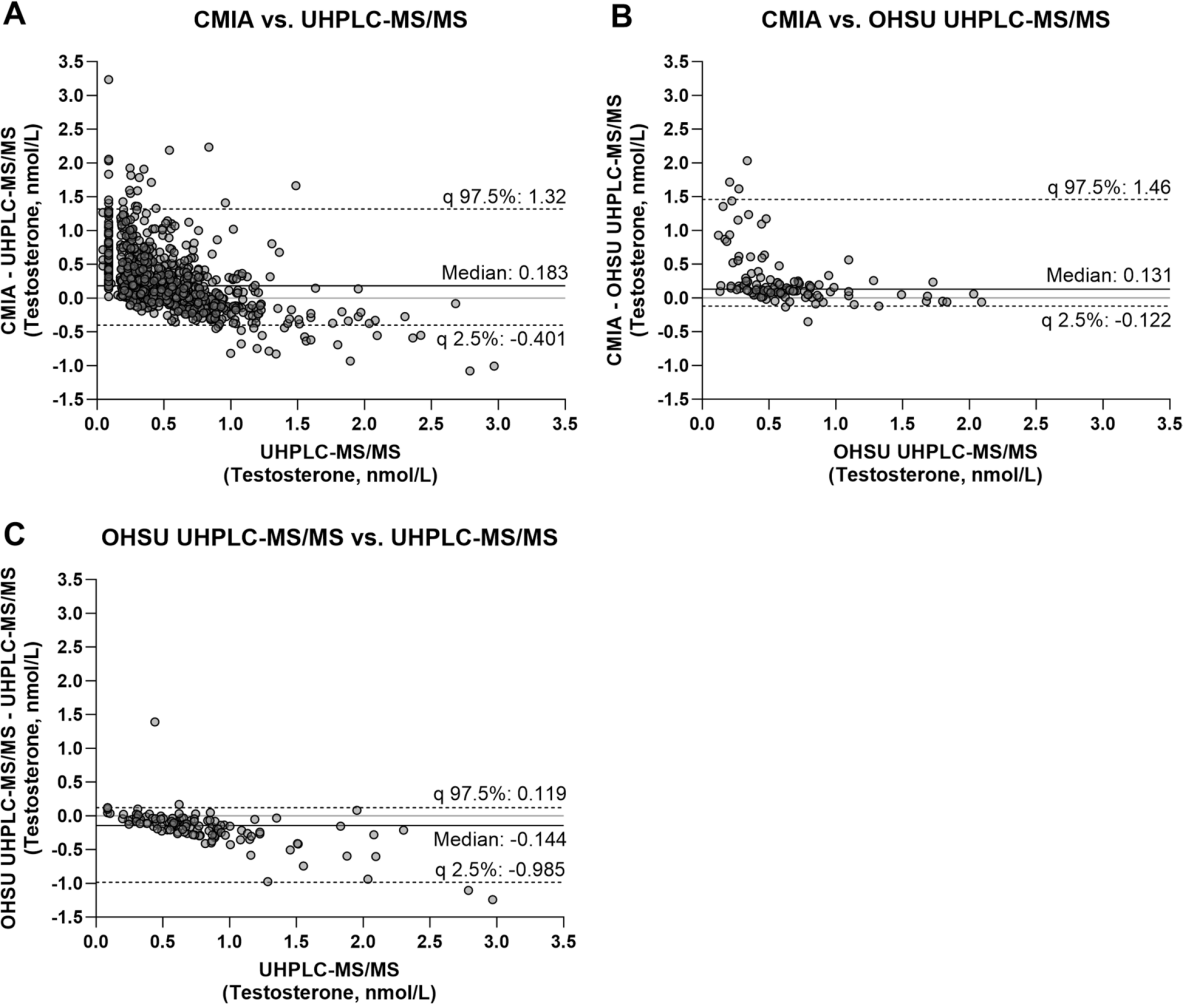
Bland–Altman plots for assessing differences in testosterone measurements determined by CMIA and each of the two MS methods (**A** – **B**), as well as between the two MS methods (**C**). In each plot, the y-axis represents the difference between serum testosterone concentrations (nmol/L) for the (**A**) CMIA minus the UHPLC-MS/MS method, (**B**) CMIA minus the OHSU UHPLC-MS/MS method and (**C**) OHSU UHPLC-MS/MS method minus the UHPLC-MS/MS method plotted against the testosterone concentration (nmol/L) obtained by UHPLC-MS/MS (**A** and **C**) or OHSU UHPLC/MS/MS (**B**) on the x-axis. Available testosterone values for participants at D0 and 25 W in both the DMPA-IM and NET-EN arms were used. The solid light grey line represents the 0% line of identity, while the solid black line represents the median difference (BIAS) between the methods and the dashed black lines represent the limits of agreement (2.5% and 97.5% quantiles). NET-EN—norethisterone enanthate; DMPA-IM—depo medroxyprogesterone acetate intramuscular; D0 – day 0; 25 W – 25 weeks; CMIA—chemiluminescent microparticle immunoassay; OHSU—Oregon Health & Science University; UHPLC-MS/MS—ultra-high performance liquid chromatography tandem mass spectrometry

**Fig. 2:**
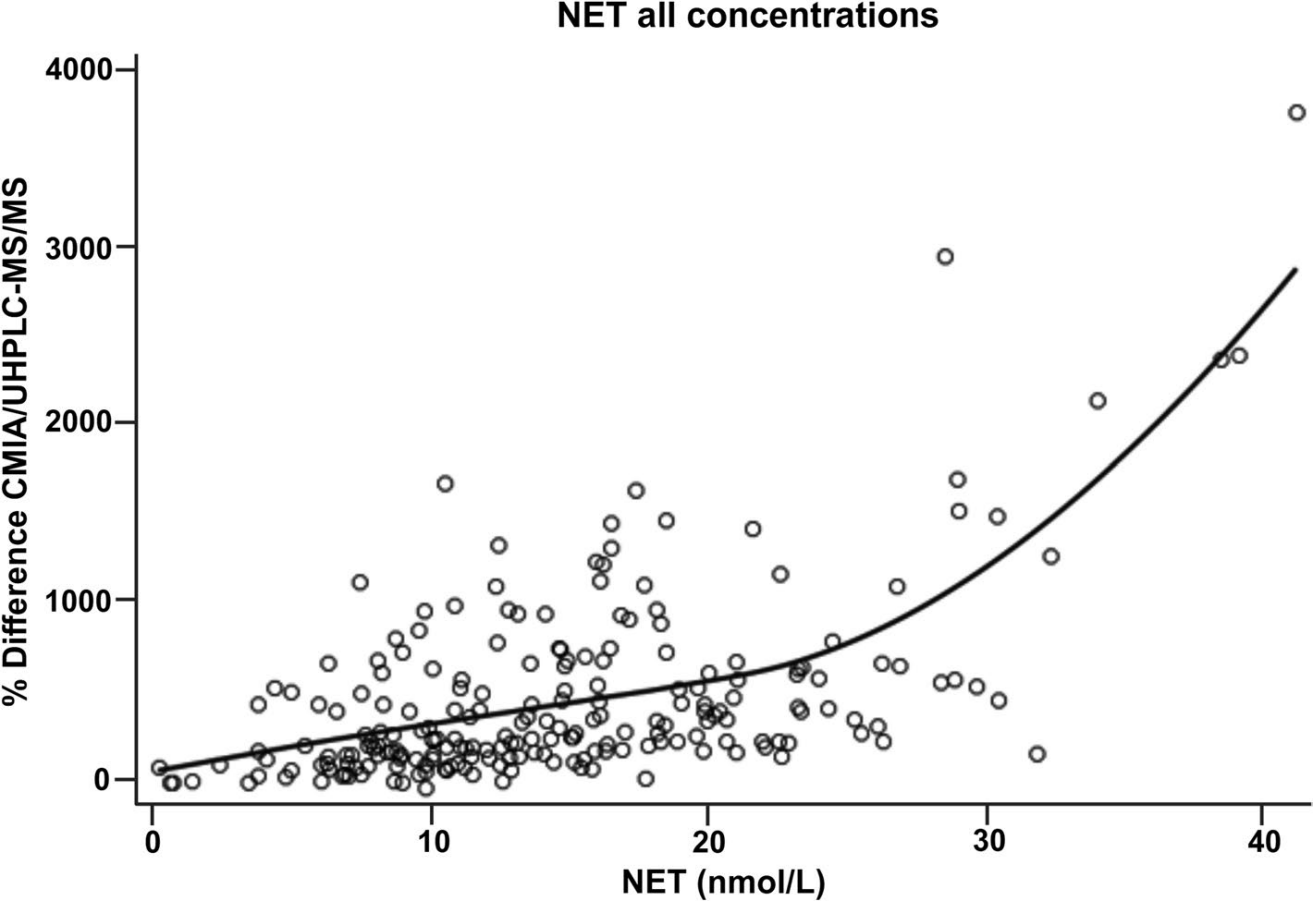
Increased NET concentrations are associated with increased concentration differences determined by CMIA vs UHPLC-MS/MS. Percentage difference in testosterone levels measured by CMIA relative to UHPLC-MS/MS [12] are plotted against donor-matched serum NET concentrations for all NET concentrations [10]**.** Local weighted regression was used to fit a smooth curve through the data points. NET-EN—norethisterone enanthate; CMIA—chemiluminescent microparticle immunoassay; UHPLC-MS/MS—ultra-high performance liquid chromatography tandem mass spectrometry

The lower limit of quantification (LLOQ) for UHPLC-MS/MS was 0.173 nmol/L [12], while the LLOQ for OHSU UHPLC-MS/MS was 0.03 nmol/L. In the CMIA data analysis, only 1 sample (25W) fell below the LOQ and was therefore not included in the analysis. Testosterone concentration percentage difference between the data obtained with CMIA vs UHPLC-MS/MS (% Difference CMIA/UHPLC-MS/MS) for each donor was calculated using the following equation: % Difference CMIA/UHPLC-MS/MS = (CMIA – UHPLC-MS/MS)/UHPLC-MS/MSX100. All the data were not normally distributed, as determined by the Shapiro–Wilk test, and hence only non-parametric tests were performed. Bland–Altman analyses were conducted to assess bias (median), along with the lower and upper limits of agreement (2.5% and 97.5% quantiles) and their respective 95% bootstrap confidence intervals (CIs). In addition, non-parametric regression techniques—including Passing-Bablok regression to evaluate the relationship between CMIA and UHPLC-MS/MS, and locally weighted regression to explore the association between NET concentrations and the differences in measurements by CMIA versus UHPLC-MS/MS—were performed using Statistical Analysis Software (SAS® V9.4, SAS Institute, Inc., Cary, NC, USA) and R (R Core Team, 2024) (Figs. 1 and 2 and Supplementary Figure S1). All other figures and statistical analyses were performed using GraphPad Prism 9.31 from GraphPad Software, Inc. (La Jolla, California, USA) (Tables 3 – 4 and Supplementary Figures S2 – S3). Statistical analysis was performed using the Kruskal–Wallis test with Dunn’s multiple comparisons (Supplementary Figures S3) or the Wilcoxon matched-pairs signed rank test (Table 4).

## Results

We quantified serum testosterone concentrations for the whole WHICH cohort (432 women) using CMIA. We first compared donor-matched serum testosterone concentrations determined by CMIA with those previously obtained by UHPLC-MS/MS in South Africa [12]. In addition, for a small subset (n = 58) of women, testosterone concentrations were also determined by OHSU UHPLC-MS/MS, in an independent laboratory in the U.S.A., and compared to the testosterone concentrations determined by CMIA and UHPLC-MS/MS. Bland–Altman analysis indicated that the testosterone concentrations determined by CMIA were different from those obtained by UHPLC-MS/MS (Bias 0.183) and OHSU UHPLC-MS/MS (Bias 0.131) (Fig. 1A and B, Table 1). While a difference in the testosterone concentrations determined by OHSU UHPLC-MS/MS and UHPLC-MS/MS was also detected (Bias −0.144), the range between the upper and lower limit of agreement (0.119 and −0.985, respectively) is much narrower than the corresponding range for the CMIA method compared to either of the two MS/MS methods (Fig. 1 and Table 1).

**Table 1:** Bland–Altman analysis of testosterone concentrations determined by CMIA and two different MS/MS methods

	CMIA vs UHPLC-MS/MS	CMIA vs OHSU UHPLC-MS/MS	OHSU UHPLC-MS/MS vs UHPLC-MS/MS
n-value*	432	58	57
Bias, Median (95% Cl)**	0.183 (0.156; 0.213)	0.131 (0.103; 0.166)	−0.144 (−0.191; −0.111)
Lower limit of agreement (95% Cl)**	−0.401 (−0.554; −0.351)	−0.122 (−0.351; −0.061)	−0.985 (−1.241; −0.597)
Upper limit of agreement (95% Cl)**	1.32 (1.225; 1.550)	1.46 (1.102; 1.747)	0.119 (0.046; 0.353)

*CMIA* Chemiluminescent microparticle immunoassay, *OHSU *Oregon Health & Science University, *UHPLC-MS/MS *Ultra-high performance liquid chromatography tandem mass spectrometry

^*^Available testosterone concentrations at D0 and 25 W in the NET-EN and DMPA-IM were compared, using testosterone values obtained from the same participants

**The 95% CIs were constructed using 5,000 bootstrap samples

Having shown that the variation between the two MS/MS methods is less than the variation between either of the MS/MS methods and the CMIA assay, we next investigated the difference between the CMIA and the UHPLC-MS/MS methods in more detail. Bland–Altman analysis showed that the percentage difference in testosterone concentrations determined by the CMIA and the UHPLC-MS/MS method at baseline (D0) in both arms together (NET-EN and DMPA-IM) is 22.2% (Table 2 and Supplementary Figure S1). Similarly, the percentage difference at D0 in the NET-EN and DMPA-IM arms is 22.2% and 23.0%, respectively (Table 2 and Supplementary Figure S1). This difference in testosterone concentrations at D0 is most apparent at testosterone concentrations less than 1 nmol/L (Supplementary Figure S1). At 25 W, there was a substantial 271% difference between the two methods in the NET-EN arm. In contrast, only a relatively small 19.3% difference was detected at 25 W in the DMPA-IM arm, which resembles those obtained at D0 (Table 2 and Supplementary Figure S1).

**Table 2:** Bland–Altman and Passing-Bablok regression of CMIA and UHPLC-MS/MS

		**Bland–Altman**	**Passing-Bablok regression**	
**Populations**	**n-value**	% Difference* (95% Cl)**	**Intercept** **(95% Cl)**	**Slope** **(95% Cl)**
NET-EN + DMPA-IM D0	432	22.2 (15.9; 29.9)	0.189 (0.14; 0.217)	0.872 (0.796; 0.961
NET-EN D0	218	22.2 (11.8; 30.2)	0.148 (0.796; 0.198)	0.901 (0.791; 1.04)
DMPA-IM D0	214	23.0 (13.6; 37.4)	0.227 (0.155; 0.283)	0.839 (0.731; 0.961)
NET-EN 25W	218	271 (229; 365)	−0.132 (−0.362; 0.230)	3.75 (2.78; 5.33)
DMPA-IM 25W	214	19.3 (11.2; 26.0)	0.241 (0.202; 0.281)	0.598 (0.504; 0.694)

*NET-EN *Norethisterone enanthate, *DMPA-IM *Depo medroxyprogesterone acetate intramuscular, *D0 *Day 0, * 25 W *25 weeks, *CMIA *Chemiluminescent microparticle immunoassay, *UHPLC-MS/MS *Ultra-high performance liquid chromatography-tandem mass spectrometry

^*^% Difference or BIAS (median) from Bland–Altman analysis (Figure S1)

**The 95% CIs were constructed using 5 000 bootstrap samples

Passing-Bablok regression line intercepts in the D0 NET-EN + DMPA-IM, D0 NET-EN, and D0 DMPA-IM populations were slightly above zero, and none of the 95% CIs spanned zero (Table 2). Additionally, for these D0 populations, the Passing-Bablok regression line slopes were close to one. Taken together, this suggests minimal unit (nmol/L) differences in testosterone values determined by the CMIA and UHPLC-MS/MS methods at D0. In the DMPA-IM arm at 25 W, the regression line intercept was comparable to those obtained in the D0 samples, but the slope (0.598) indicates that at higher testosterone concentrations, the UHPLC-MS/MS method slightly overestimated the testosterone concentrations. In the NET-EN arm at 25 W, the regression line intercept was close to zero, but the slope of 3.75 (2.78; 5.33) indicates that the CMIA assay substantially overestimated testosterone concentrations.

Interestingly, while the Bland–Altman and Passing-Bablok regression analysis suggest only a small unit difference in testosterone measurements at D0, in 50.92% and 51.87% of the samples in the NET-EN and DMPA-IM arms, respectively, the testosterone concentrations determined by CMIA were > 20% higher than those determined by UHPLC-MS/MS in matching samples (Supplementary Figure S2). Similarly, at 25 W, 49.77% of the participants in the DMPA-IM arm had CMIA concentrations > 20% higher than those determined by UHPLC-MS/MS, while almost all (94.7%) of the participants in the NET-EN arm had CMIA concentrations > 20% higher than those determined by UHPLC-MS/MS (Supplementary Figure S2).

Having shown that the greatest difference between the CMIA and UHPLC-MS/MS testosterone concentrations occurs in the 25 W NET-EN samples, we next attempted to quantify the NET interference with the CMIA assay. For this, we calculated the % difference between the two methods (% Difference CMIA/UHPLC-MS/MS), as described in the Methods. When plotting the % difference in testosterone concentrations with matched NET concentrations, local weighted regression analysis indicated that NET dose-dependently increased the % difference in testosterone concentrations obtained between the two methods (Fig. 2).

The median % difference in testosterone concentrations at D0 (for the whole cohort) was 22.0% (Table 3). Estimates obtained from local weighted regression analysis (Fig. 2), indicated that there is a 182, 309, 428, and 550% difference in testosterone concentrations between the CMIA and UHPLC-MS/MS methods at 5.00, 10.0, 15.0, and 20.0 nmol/L NET, respectively (Table 3).

**Table 3:** Percentage difference in testosterone concentrations between CMIA and UHPLC-MS/MS methods at different NET concentrations

NET concentration (nmol/L)	% Difference in testosterone concentrations: CMIA/UHPLC-MS/MS^#^
0.00*	22.0 (15.9; 30.3)
5.00	181 (71.8; 292)
10.0	309 (246; 372)
15.0	428 (360; 497)
20.0	550 (470; 630)

*NET-EN *Norethisterone enanthate, *CMIA *Chemiluminescent microparticle immunoassay, *UHPLC-MS/MS *Ultra-high performance liquid chromatography-tandem mass spectrometry

^*^For 0.00 nmol/L, the median (95% CI) % difference in testosterone concentrations: CMIA/UHPLC-MS/MS was calculated for baseline (D0) samples from the whole cohort (n = 432 participants)

^#^Estimates used for NET calculations at 5.00–20.0 nmol/L are from Fig. 2 local weighted regression

We next compared donor-matched testosterone concentrations for the whole cohort at D0 and 25 W for both contraceptive arms, determined by CMIA and UHPLC-MS/MS [12], and found that the testosterone concentrations measured by the CMIA method were significantly higher than those measured by UHPLC-MS/MS for all conditions except in the DMPA-IM arm at 25 W (Table 4). The median testosterone concentrations at D0 in the NET-EN and DMPA-IM arms were significantly higher with the CMIA (0.650 and 0.700 nmol/L, respectively) compared to the UHPLC-MS/MS (0.551 and 0.562 nmol/L, respectively) method (Table 4). However, at 25 W, the median testosterone concentration in the NET-EN arm was significantly and substantially higher with the CMIA compared to the UHPLC-MS/MS method (0.975 vs 0.253 nmol/L) (Table 4). Conversely, no significant difference in the median testosterone concentrations was detected between the CMIA and the UHPLC-MS/MS methods in the DMPA-IM arm at 25 W (0.490 vs 0.423 nmol/L) (Table 4), and both methods indicated a significant decrease in testosterone concentrations from D0 to 25 W for the DMPA-IM arm (Supplementary Figure S3), consistent with our previous findings [12]. However, in the NET-EN arm, the CMIA method indicated a significant increase in testosterone concentrations from D0 to 25 W (Supplementary Figure S3), which is inconsistent with the significant decrease previously reported with the UHPLC-MS/MS method [12].

**Table 4:** Median levels of serum testosterone measured by CMIA and UHPLC-MS/MS

	**CMIA**		**UHPLC-MS/MS**		**CMIA vs UHPLC-MS/MS**
**Cohort**	**n**	**Median** (95% Cl)*	**n**	**Median** (95% Cl)*	*p*-value**
NET-EN D0	218	0.650 (0.600; 0.740)	218	0.551 (0.489; 0.596)	**0.0081**
NET-EN 25W	218	0.975 (0.900; 1.05)	218	0.253 (0.239; 0.277)	**< 0.0001**
DMPA-IM D0	214	0.700 (0.650; 0.780)	214	0.562 (0.517; 0.624)	**< 0.0001**
DMPA-IM 25W	214	0.490 (0.470; 0.510)	214	0.418 (0.378; 0.465)	> 0.999

*CI *Confidence interval, *NET-EN *Norethisterone enanthate, *DMPA-IM *Depo medroxyprogesterone acetate intramuscular, *D0 *Day 0, * 25 W *25 weeks, *CMIA *Chemiluminescent microparticle immunoassay, *UHPLC MS/MS *Ultra-high performance liquid chromatography-tandem mass spectrometry

^*^Median values in nmol/L

**For ease of reference, select p-values generated in Supplementary Material S3 using the Kruskal–Wallis test with Dunn’s multiple comparisons post-test, are also indicated in this table

## Discussion

Ours is the first study showing that physiological concentrations of NET in serum from NET-EN users, dose-dependently and substantially interfere with testosterone measurements by a CMIA method (the Abbott Architect 2nd generation CMIA). In the package insert [25], it is suggested that a testosterone concentration of 2.4 nmol/L is ~ 3% higher in the presence of 10 nmol/L NET. The median testosterone concentration in our cohort at baseline is only 0.680 nmol/L or 0.555 nmol/L (as determined by CMIA or UHPLC-MS/MS, respectively), making it difficult to directly compare our results with those of the package insert (Supplementary Table S1). However, using donor matched UHPLC-MS/MS testosterone reference values [12] and NET concentrations [10], we show that at 5 and 10 nmol/L NET, the Abbott Architect CMIA method results in 143% and 297%, respectively, higher testosterone values than the UHPLC-MS/MS method (Table 3). Our results show a dose-dependent increase in error (% Difference) for testosterone concentrations with increasing NET concentrations. We also show that the degree of interference by 10 nmol/L NET is much greater than 3% at low testosterone concentrations and that substantial interference occurs at concentrations of NET lower than 10 nmol/L.

Of particular relevance for the interpretation of the biological effects of DMPA-IM and NET-EN is the finding of a 271% difference in testosterone values obtained by CMIA (median testosterone 0.975 nmol/L) compared to those obtained by UHPLC-MS/MS (median testosterone 0.253 nmol/L) in our 25 W NET-EN arm (Tables 2 and 4). These results falsely show a significant increase in median testosterone concentrations from D0 to 25 W (0.650 to 0.975 nmol/L, p < 0.0001) with the CMIA method (Table 4 and Supplementary Figure S3), compared to the decrease in median testosterone concentration that has previously been reported for the UHPLC-MS/MS method (0.551 to 0.253 nmol/L, p < 0.0001) in the NET-EN arm (Table 4 and Supplementary Figure S3; [12]). The high degree of NET interference with the CMIA testosterone measurements, therefore, not only results in falsely elevated testosterone values but also confounds the interpretation of the effects of NET-EN contraception on testosterone concentrations, as well as the relative effects of DMPA-IM compared to NET-EN. For the DMPA-IM arm at 25 W, we did not see a significant difference between the median testosterone concentrations determined by the two methods, indicating that MPA does not interfere with the CMIA method (Table 4). The significant decrease in testosterone concentrations from D0 to 25 W for the CMIA method is consistent with our previous findings using the UHPLC-MS/MS method, showing that testosterone concentrations are decreased in women using DMPA-IM [12].

The median testosterone concentration at baseline in this cohort of women (NET-EN + DMPA-IM D0) was 0.680 (95% CI: 0.640–0.730) or 0.555 (95% CI: 0.527–0.586) nmol/L as determined by the Abbott CMIA or UHPLC-MS/MS method, respectively (Supplementary Table S1), which falls within the normal range for healthy adult women, ranging from 0.06–1.68 nmol/L [20–23]. Interestingly, for all the baseline comparisons (the whole cohort (NET-EN + DMPA-IM D0), NET-EN D0, and DMPA-IM D0), the testosterone concentrations determined by the CMIA method were higher (22.2, 22.2, and 23.0%, respectively) than those of the UHPLC-MS/MS method (Table 2). A possible explanation for the small difference in baseline testosterone concentrations between the two methods is NET interference with the CMIA assay. Although trial participants self-reported no NET-EN use four months before trial initiation, low levels of NET (0.246–0.348 nmol/L) were detected at baseline in serum from a substantial number (28.5%) of trial participants [10]. However, comparing the testosterone concentrations determined by the two methods in only those participants where no NET was detected at baseline, a significant difference was still observed (Supplemental Table S1), indicating that non-study NET is not the cause of the difference in baseline testosterone concentrations between the two methods. Given that our UHPLC-MS/MS method also had a similar LLOQ [12] to the reported LOD for the Abbott Architect method, the differences in baseline data between methods are potentially due to interference by endogenous androgens. These could include DHEA-S, the most abundant androgen in circulation, as well as other androgens structurally related to testosterone [25–27]. Additionally, a recent paper suggests that the concentration of testosterone in the standards supplied for at least one immunoassay may be incorrect as assessed by UPLC-MS/MS [36], providing another explanation for the difference in testosterone concentration we observe in our D0 samples.

There is only one study, to our knowledge, that determined testosterone concentrations in women using NET-EN [15]. Using a Chiron Diagnostics CMIA to measure testosterone concentrations in the serum of women six weeks after receiving one 200 mg NET-EN (n = 72) or placebo (n = 61) injection, Lawrie et al*.* found no significant difference in total testosterone concentrations (mean 1.08 and 1.52 nmol/L for NET-EN and placebo, respectively). It is noteworthy that while the mean testosterone concentrations in the Lawrie et al. study (NET-EN arm 1.08 nmol/L) are very similar to those reported here for CMIA (25W NET-EN arm 0.975 nmol/L), these concentrations are very different to, and significantly higher than, the concentrations determined by UHPLC-MS/MS (25W NET-EN 0.253 nmol/L; [12]), consistent with NET interference.

To our knowledge, this is the first study to report on the extent of NET interference using the Abbott Architect 2nd generation testosterone CMIA method in serum samples from women receiving NET-EN injectable contraception, and in a relatively large cohort of women. Our results are in agreement with a study by Jeffery et al., on interference by orally administered NET in 3 women [28], but indicate a greater degree of NET interference with the Abbott CMIA. Our results are also consistent with the study by Rowe and Rabet that showed exogenously added NET (10, 50, and 100 nmol/L) dose-dependently increased interference with the Abbott Architect CMIA [29].

PCOS is commonly, but not always, associated with increased testosterone concentrations in women [23, 37]. A limitation of our study is that we do not know whether any of the participants had previously been diagnosed with PCOS. Another limitation of our study is that we only investigated NET interference with the Abbott Architect CMIA method and did not determine possible NET interference with other CMIA methods, which may have shown different results. We also did not investigate potential interference by NET for the determination of other endogenous steroids structurally related to testosterone in our samples, due to limited sample volumes. It was beyond the scope of this study to investigate whether progestins other than NET and MPA interfere with the determination of serum testosterone levels in premenopausal women on contraception. Interestingly, the progestin levonorgestrel (LNG), which is widely used in contraceptive implants, intrauterine devices, or in combined oral contraceptives [9], has a chemical structure similar to that of testosterone and NET (Supplementary Figure S4). Norgestrel, the non-biologically active form of LNG, reportedly interferes at 20 ng/mL (64 nmol/L) with 2.4 nmol/L testosterone [25]. LNG concentrations as high as 58.2 nmol/L have been reported in women using LNG-containing combined oral contraception [38], suggesting that some interference with the Abbott Architect CMIA testosterone assay is possible. Using the Abbott Architect CMIA testosterone assay, Hofmeyr et al*.* recently showed that the LNG subdermal implant and DMPA-IM reduced total testosterone levels to a similar degree 6 months after initiation [13]. While this study did not report LNG serum concentrations in their cohort, these have previously been reported to be 0.4–1.3 nmol/L one month – 6 years after insertion [9], making it unlikely that the LNG would have interfered with the Abbott Architect CMIA, although this remains to be established. NET and LNG are also used in menopausal hormone therapy in postmenopausal women. However, the low daily oral dose (NET 1 mg (3.35 mol) and LNG 0.150–0.250 mg (0.480–0.800 mol)) (reviewed in [39]) is also unlikely to interfere with the Abbott Architect CMIA.

## Conclusions

In conclusion, the high degree of NET interference in the Abbott Architect testosterone CMIA at physiological concentrations of testosterone and NET in NET-EN users confounded the biological outcome of NET-EN use on testosterone concentrations. We suggest that immunoassays should be avoided when measuring testosterone in clinical samples from all premenopausal women. This is of particular importance for women on contraception, since several progestins structurally related to testosterone and used in contraception may interfere. This is in agreement with proposals to only use sensitive, specific, and accurate LC–MS/MS methods for clinical research on sex steroids [40], as well as in hospitals and national laboratories in Spain, Korea, and the UK [16, 41, 42]. However, LC–MS/MS instrumentation is expensive and requires highly trained operators, and consequently, not all hospitals and national laboratories, especially in sub-Saharan Africa, can implement this technology. As such, the limitations of immunoassays for the use of testosterone measurement in women, especially those on contraception, should be better defined, and the results obtained by immunoassays should be interpreted with caution.

## Supplementary Information

Additional file 1: Supplementary Table and Figures. Supplementary Table S1: Median concentrations of testosterone at D0 in samples with or without detectable concentrations of NET. Supplementary Figure S1: Bland-Altman plots for assessing differences in testosterone concentrations determined by CMIA and UHPLC-MS/MS at baselineand 25 weeks. Supplementary Figure S2: Percentage under- and overestimation of testosterone concentrations by CMIA compared to UHPLC-MS/MS in paired samples. Supplementary Figure S3: In NET-EN users, testosterone concentrations measured by CMIA are higher than those measured by UHPLC-MS/MS. Supplementary Figure S4: Chemical structures of testosterone, norethisterone, medroxyprogesterone, DHEA-S, 19-nortestosterone and levonorgestrel

## Data Availability

The datasets used and/or analyzed during the current study are available from the corresponding author on reasonable request.

## Abbreviations

DMPA-IM: Intramuscular depo medroxyprogesterone acetate
NET-EN: Norethisterone enanthate
WHICH: Women’s Health Injectable Contraceptive and HIV
D0: Baseline
25W: 25 Weeks
UHPLC-MS/MS: Ultra-high performance liquid chromatography tandem mass spectrometry
DMPA-SC: Sub-cutaneous DMPA
CMIA: Chemiluminescent microparticle immunoassay
LC–MS/MS: Liquid chromatography-tandem mass spectrometry
NET: Norethisterone/norethindrone
MPA: Medroxyprogesterone acetate
DHEA-S: Dehydroepiandrosterone sulphate
LOQ: Limit of quantification
LLOQ: Lower limit of quantification
OHSU: Oregon Health & Science University
PCOS: Polycystic ovary syndrome
