# Supplementary material for: Serum norethisterone (NET) levels in NET-enanthate (NET-EN) injectable contraception users substantially interfere with testosterone immunoassay measurements and confound interpretation of biological outcomes
Source: Contracept Reprod Med. 2025 Aug 19;10:51. doi: 10.1186/s40834-025-00388-x (PMC12363054; doi:10.1186/s40834-025-00388-x)
Supplement: Supplementary file 1 — Additional file 1: Supplementary Table and Figures. Supplementary Table S1: Median concentrations of testosterone at D0 in samples with or without detectable concentrations of NET. Supplementary Figure S1: Bland-Altman plots for assessing differences in testosterone concentrations determined by CMIA and UHPLC-MS/MS at baselineand 25 weeks. Supplementary Figure S2: Percentage under- and overestimation of testosterone concentrations by CMIA compared to UHPLC-MS/MS in paired samples. Supplementary Figure S3: In NET-EN users, testosterone concentrations measured by CMIA are higher than those measured by UHPLC-MS/MS. Supplementary Figure S4: Chemical structures of testosterone, norethisterone, medroxyprogesterone, DHEA-S, 19-nortestosterone and levonorgestrel [file 40834_2025_388_MOESM1_ESM.docx]

**Supplementary Table and Figures**

**Table S1. Median concentrations of testosterone at D0 in samples with or without detectable concentrations of NET.**

| **Samples** | **CMIA** | **UHPLC-MS/MS** | **CMIA vs UHPLC-MS/MS** |
| --- | --- | --- | --- |
|  | **Median**  **(95% CI)** | **Median**  **(95% CI)** | **p-value** |
| All (NET-EN + DMPA-IM) | 0.680  (0.640; 0.730) | 0.555  (0.527; 0.586) | **p < 0.0001** |
| No detectable NET | 0.730  (0.650; 0.780) | 0.558  (0.503; 0.614) | **p < 0.0001** |
| Only with detectable NET | 0.635  (0.590; 0.690) | 0.551  (0.517; 0.593) | **0.0015** |

Median values in nmol/L; All (NET-EN + DMPA-IM), No detectable NET and Only with detectable NET indicate D0 samples from the whole cohort, D0 samples with no detectable non-study NET, and only the D0 samples with detectable non-study NET, respectively (level of detection for NET: 0.0838 nmol/L (Avenant, Bick et al. 2023)). Statistical significance was determined using Wilcoxon matched-pairs signed rank test; CI – Confidence interval; NET-EN - norethisterone enanthate; DMPA-IM - depo-medroxyprogesterone acetate intramuscular; D0 – day 0; CMIA - chemiluminescent microparticle immunoassay; UHPLC-MS/MS - ultra-high performance liquid chromatography tandem mass spectrometry.


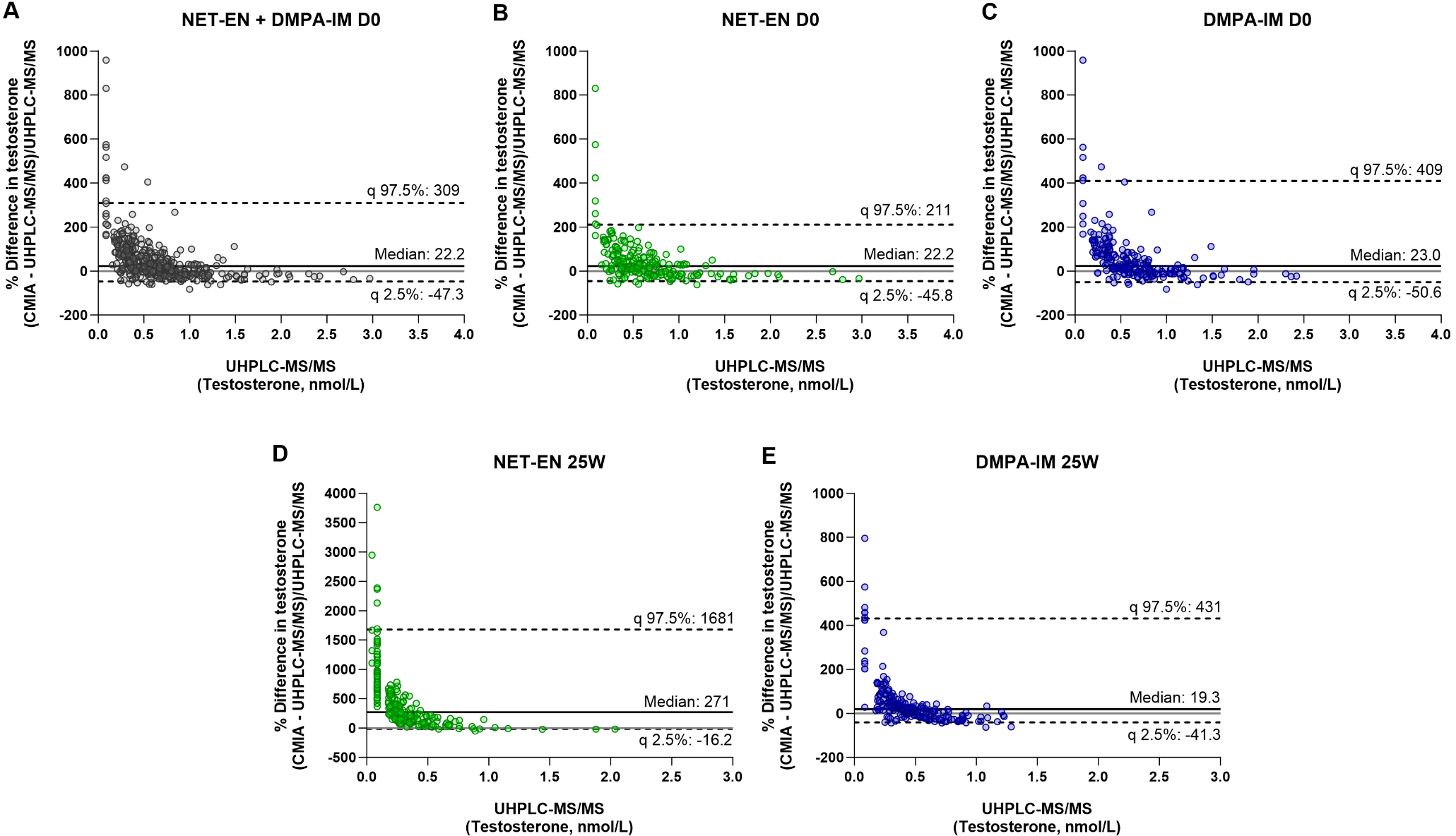


**Figure S1. Bland-Altman plots for assessing differences in testosterone concentrations determined by CMIA and UHPLC-MS/MS at baseline (A – C) and 25 weeks (D – E).** In each plot, the y-axis represents the percentage difference in serum testosterone concentrations (CMIA minus UHPLC-MS/MS) plotted against the concentration of testosterone (nmol/L) as determined by UHPLC-MS/MS on the x-axis. Available testosterone values for participants at D0 in (A) both the NET-EN and DMPA-IM arm, (B) the NET-EN arm, (C) the DMPA-IM arm and at 25W in (D) the NET-EN arm or (E) the DMPA-IM arm. The solid light grey line represents 0% line of identity, while the solid black line represents the median difference (BIAS) between the methods and the dashed black lines represent the limits of agreement (2.5% and 97.5% quantiles). NET-EN - norethisterone enanthate; DMPA-IM - depo-medroxyprogesterone acetate intramuscular; D0 – day 0; 25W – 25 weeks; CMIA - chemiluminescent microparticle immunoassay; UHPLC-MS/MS - ultra-high performance liquid chromatography tandem mass spectrometry.

**Figure S2. Percentage under- and overestimation of testosterone concentrations by CMIA compared to UHPLC-MS/MS in paired samples.** Serum samples collected from women randomized to NET-EN or DMPA-IM at D0 and 25W were assayed for total testosterone (nmol/L) using CMIA and compared to those obtained by UHPLC-MS/MS (Avenant, Singata-Madliki et al. 2024) from the same women. The CMIA/UHPLC testosterone ratio for each individual was calculated and stratified into 3 groups: (1) Testosterone concentration determined by CMIA was < 20% of corresponding testosterone concentration determined by UHPLC-MS/MS (thus CMIA/UHPLC-MS/MS ratio < 0.8); (2) testosterone concentration determined by CMIA > 20% of corresponding testosterone concentration determined by UHPLC-MS/MS (thus CMIA/UHPLC-MS/MS ration > 1.2) and (3) those in between (-20 to 20%). NET-EN - norethisterone enanthate; DMPA-IM - depo-medroxyprogesterone acetate intramuscular; D0 – day 0; 25W – 25 weeks.


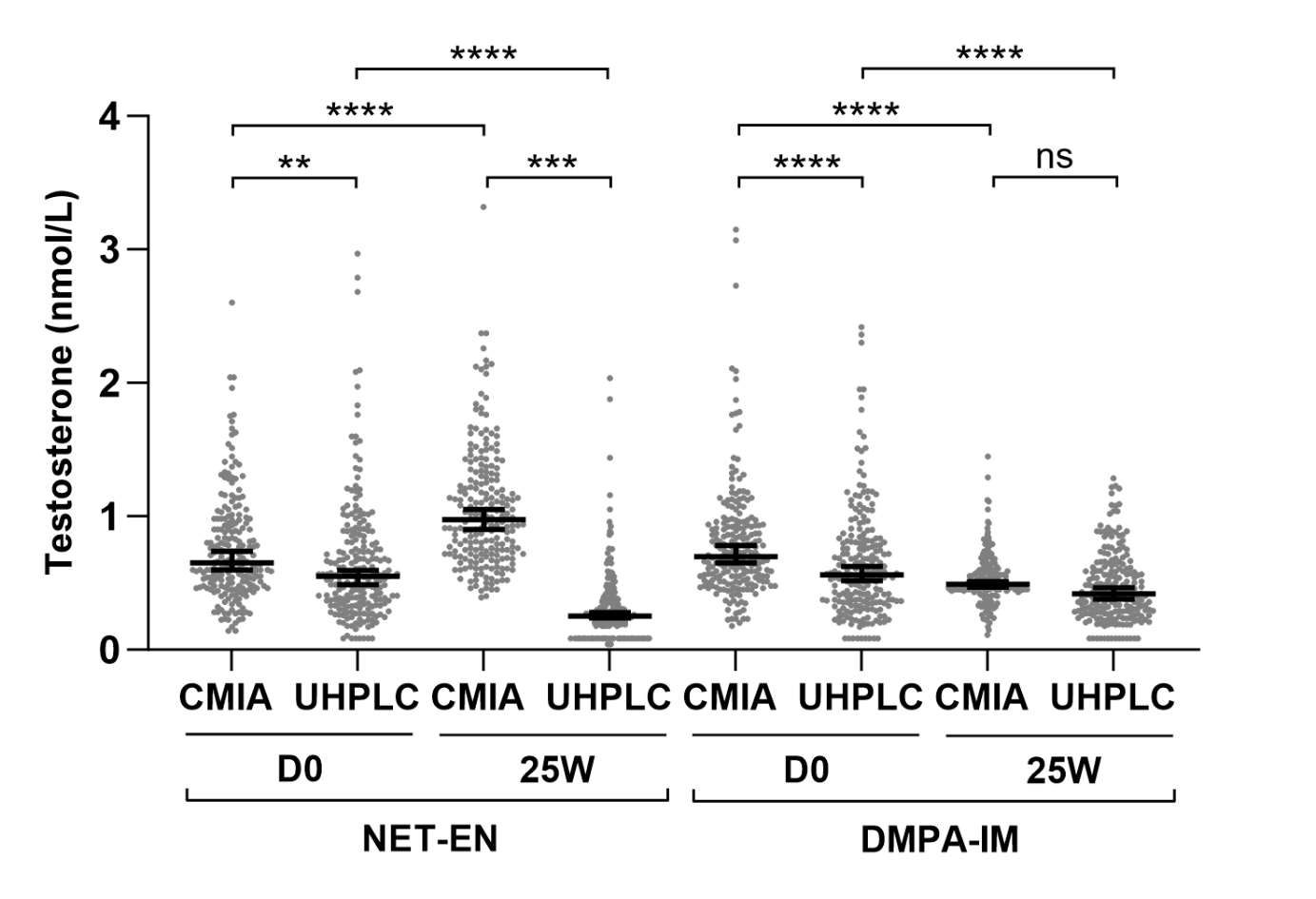


**Figure S3. In NET-EN users, testosterone concentrations measured by CMIA are higher than those measured by UHPLC-MS/MS.** Serum samples collected from women randomized to NET-EN or DMPA-IM at D0 and 25W were assayed for total testosterone using CMIA and compared to those obtained by UHPLC-MS/MS on the same women (12). Graphs indicate median with 95% confidence intervals with sample sizes shown in Table 4. Statistical analysis was performed using the Kruskal-Wallis test with Dunn’s multiple comparisons post-test. Statistical significance is denoted as * and **** to indicate p<0.05 or p<0.0001, respectively, while ns indicates no statistical significance. For aesthetic reasons, not all possible statistical comparisons are shown on the graph and UHPLC-MS/MS is shown as UHPLC. NET-EN - norethisterone enanthate; DMPA-IM - depo-medroxyprogesterone acetate intramuscular; D0 – day 0; 25W – 25 weeks; CMIA - chemiluminescent microparticle immunoassay; UHPLC-MS/MS - ultra-high performance liquid chromatography tandem mass spectrometry.


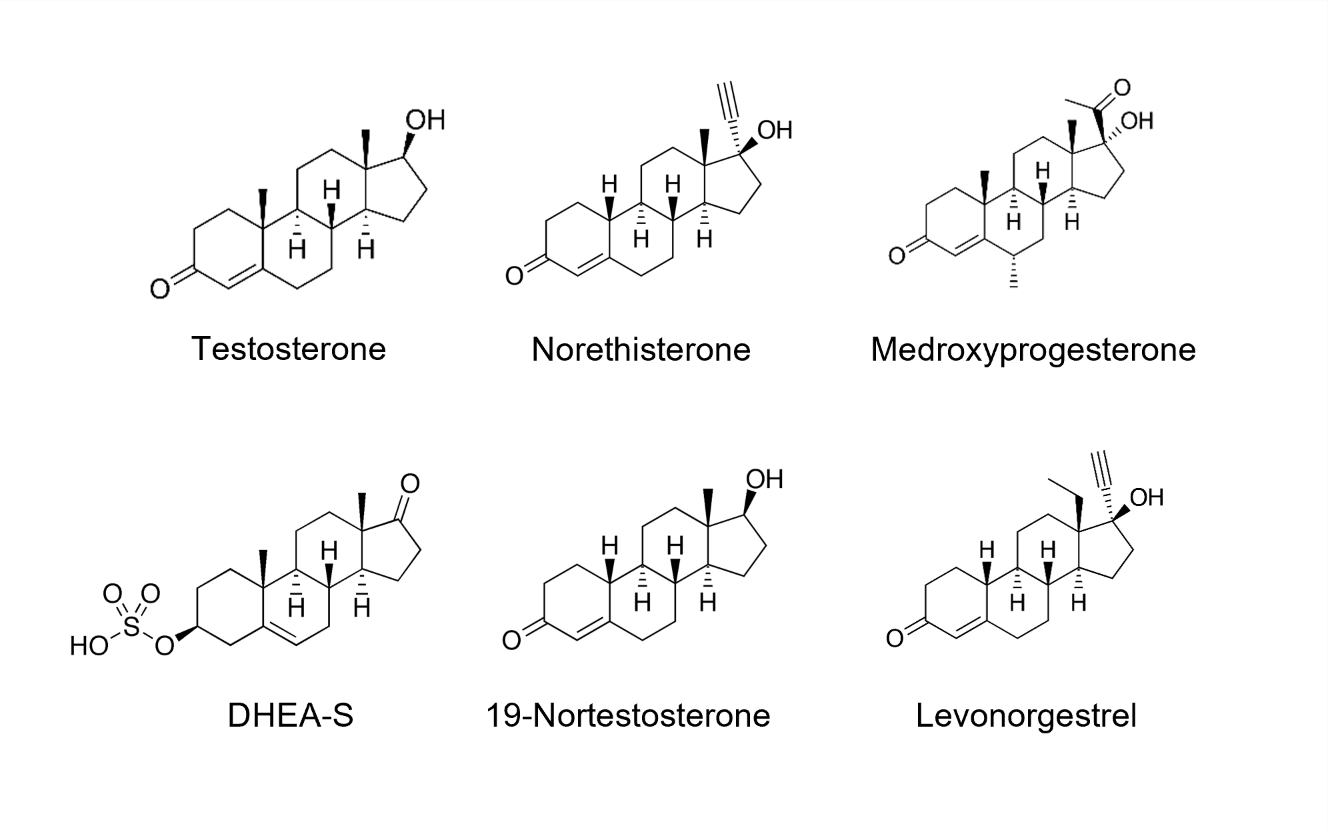


**Figure S4.** **Chemical structures of testosterone, norethisterone, medroxyprogesterone, DHEA-S, 19-nortestosterone and levonorgestrel.**

Avenant, C., A. J. Bick, S. B. Skosana, S. Dlamini, Y. Balakrishna, J. M. Moliki, M. Singata-Madliki, G. J. Hofmeyr, J. Smit, M. Beksinska, I. Beesham, I. Seocharan, J. Batting, P. L. Chen, K. H. Storbeck, D. Africander and J. P. Hapgood (2023). "Misreporting contraceptive use and the association of peak study progestin levels with weight and BMI among women randomized to the progestin-only injectable contraceptives DMPA-IM and NET-EN." PLoS One 18(12): e0295959.

Avenant, C., S. Singata-Madliki, A. J. Bick, D. Africander, Y. Balakrishna, K.-H. Storbeck, M. J. M., S. Dlamini, S. B. Skosana, J. Smit, M. Beksinska, I. Beesham, I. Seocharan, J. Batting, G. J. Hofmeyr and J. P. Hapgood (2024). "The injectable contraceptives depot medroxyprogesterone acetate and norethisterone enanthate substantially and differentially decrease testosterone and sex hormone binding globulin levels: a secondary study from the WHICH randomized clinical trial." PLoS One 19(8): e0307736.
